# Hydrogels for Cardio and Vascular Tissue Repair and Regeneration

**DOI:** 10.3390/gels10030196

**Published:** 2024-03-13

**Authors:** Ilenia Motta, Michelina Soccio, Giulia Guidotti, Nadia Lotti, Gianandrea Pasquinelli

**Affiliations:** 1 Alma Mater Institute on Healthy Planet, University of Bologna, Via Massarenti 11, 40138 Bologna, Italy; ilenia.motta2@unibo.it; 2 Civil, Chemical, Environmental and Materials Engineering Department, University of Bologna, Via Terracini 28, 40131 Bologna, Italy; giulia.guidotti9@unibo.it (G.G.); nadia.lotti@unibo.it (N.L.); 3 Department of Medical and Surgical Sciences (DIMEC), University of Bologna, Via Massarenti 9, 40138 Bologna, Italy; gianandr.pasquinelli@unibo.it; 4 Pathology Unit, IRCCS Azienda Ospedaliero-Universitaria di Bologna, 40138 Bologna, Italy

**Keywords:** hydrogels, biomaterial, tissue repair, cardiovascular, aneurysm

## Abstract

Cardiovascular disease (CVD), the leading cause of death globally, affects the heart and arteries with a variety of clinical manifestations, the most dramatic of which are myocardial infarction (MI), abdominal aortic aneurysm (AAA), and intracranial aneurysm (IA) rupture. In MI, necrosis of the myocardium, scar formation, and loss of cardiomyocytes result from insufficient blood supply due to coronary artery occlusion. Beyond stenosis, the arteries that are structurally and functionally connected to the cardiac tissue can undergo pathological dilation, i.e., aneurysmal dilation, with high risk of rupture. Aneurysms of the intracranial arteries (IAs) are more commonly seen in young adults, whereas those of the abdominal aorta (AAA) are predominantly seen in the elderly. IAs, unpredictably, can undergo rupture and cause life-threatening hemorrhage, while AAAs can result in rupture, internal bleeding and high mortality rate. In this clinical context, hydrogels, three-dimensional networks of water-seizing polymers, have emerged as promising biomaterials for cardiovascular tissue repair or protection due to their biocompatibility, tunable properties, and ability to encapsulate and release bioactive molecules. This review provides an overview of the current state of research on the use of hydrogels as an innovative platform to promote cardiovascular-specific tissue repair in MI and functional recovery or protection in aneurysmal dilation.

## 1. Introduction

Hydrogels are three-dimensional polymeric structures capable of absorbing large quantities of water [1]. Due to this characteristic and their soft consistency, they can simulate living tissue; furthermore, they can be biodegradable and are stable under physiological conditions. Hydrogels can be stable, or they can degrade and eventually dissolve; in the first case, they are defined as permanent or chemical hydrogels, characterized by a network cross-linked through covalent bonds, while in the second case they are defined as reversible or physical, in which the network is held together by secondary bonds (ionic bonds, hydrogen bonds, or hydrophobic forces) [2]. Thanks to their properties (biocompatibility, swelling in aqueous environment, sensitivity to external environment stimuli), it has been possible to obtain commercial products in various fields, as in the case of the biomedical and pharmaceutical fields. Hydrogels have been studied to develop contact lenses, wound dressing, drug and gene delivery, tissue engineering, cell scaffolds, tissue repair, immunotherapies, and vaccines [3,4,5,6,7,8].

Hydrogels can be classified depending on the material used to synthesize them, they therefore are divided into natural and synthetic types. Natural hydrogels are the most biocompatible, as they are generally composed of extracellular matrix (ECM) components such as collagen, fibrin, hyaluronic acid (HA), and derivatives of natural materials, like chitosan and alginate.

Collagen is the main component of the ECM and its function is to provide mechanical support to the tissue. It has a low inflammatory response, good biocompatibility, and is biodegradable, and for these reasons many studies have been carried out using collagen alone or in combination with different materials [9].

Fibrin is a protein involved in the tissue repair process; gels composed of this protein can be considered an alternative to collagen since fibrin can be easily obtained from patients’ blood. Fibrin is widely used in cardiac tissue engineering but has low mechanical strength; therefore, studies have been carried out combining fibrin with other materials to improve the mechanical properties [9].

HA is a non-sulphated glycosaminoglycan and represents one of the main components of the ECM of skin and cartilage. HA and its derivatives have been widely used as medical products thanks to their good biocompatibility, degradability, and high hydrophilicity [10,11].

Chitosan is a partially deacetylated derivative of chitin. It has anti-bacterial properties, is easy to sterilize, is biocompatible, and its degradation can be controlled by modifying its degree of deacetylation. For these reasons, chitosan is a good material for the production of hydrogels; in fact, it has been used for tissue regeneration alone or combined with other materials [10,11,12].

Alginate is a polysaccharide derived from brown seaweed, and it has good biocompatibility, and is non-toxic and hydrophilic. It has therefore been widely used in bio-medical applications [11,12].

Overviews of natural hydrogels in tissue engineering and regenerative medicine can be found elsewhere [9,11,12]. The disadvantage of these natural hydrogels, however, is that their properties are difficult to control and lack of reproducibility.

On the contrary, synthetic hydrogels are made of synthetic polymers; in this case, the properties can be controlled and tuned more easily and are reproducible [4]. The first synthetic hydrogel that was described is poly(2-hydroxyethyl methacrylate) (PHEMA), synthesized in 1960 by Wichterle and Lim [13]. Since then, there has been a great interest in synthetic hydrogels for biomedical applications. Among the main synthetic hydrogels that have been used, there are poly(vinyl alcohol) (PVA), poly(ethylene glycol) (PEG), polycaprolactone (PCL), and poly(lactic acid) (PLA). PVA is a hydrophilic and biodegradable hydrogel produced by hydrolysis of polyvinyl acetate (PVAc) [14]. It is non-toxic, biocompatible, bio-adhesive, transparent, and therefore highly studied for biomedical and pharmaceutical applications, such as contact lenses, wound dressing, and drug delivery [15,16].

PEG is a polyether. It is hydrophilic, non-toxic, non-immunogenic, non-degradable, and has good biocompatibility. It has been studied extensively for a variety of applications, such as drug delivery, wound healing, and tissue regeneration. An advantage of using PEG for the delivery of factors and cells for tissue regeneration is that it can contain more than 95% water and therefore replicates soft tissue [17,18]. To broaden its applications and improve its properties, PEG has been combined with other types of materials, such as aliphatic polyesters [19,20,21,22,23]. Aliphatic polyesters are biodegradable polymers and are among the most used materials in biomedical applications.

PCL is a permeable biocompatible polymer with a low melting point and high biodegradability, and it is soluble in a wide range of polymers [24]. It has been used in tissue engineering and drug delivery systems [25,26,27].

PLA is a linear polymer composed of lactic acid, it has excellent biocompatibility and bio-absorbability, and it is degraded by the hydrolysis of ester bonds. It has been used for the development of tissue engineering scaffolds, delivery systems, and absorbable medical implants and sutures. A comprehensive review on PLA and its applications can be found here [28].

Hydrogels can also be semi-synthetic, and therefore composed of a combination of natural polymers and synthetic polymers. One of the most studied semi-synthetic hydrogels is gelatin methacrylate (Gel-MA), formed by gelatin, which is a natural protein obtained from collagen, and methacrylic anhydride (MA), which replaces the amino groups of gelatin with methacryloyl groups. The advantage of using Gel-MA is that the degree of methacrylation can be modified based on the application; furthermore, given the presence of gelatin, it is the ideal candidate for cell adhesion and growth. The use of Gel-MA, particularly for cardiac tissue engineering, has been covered in detail here [29].

**Figure 1:**
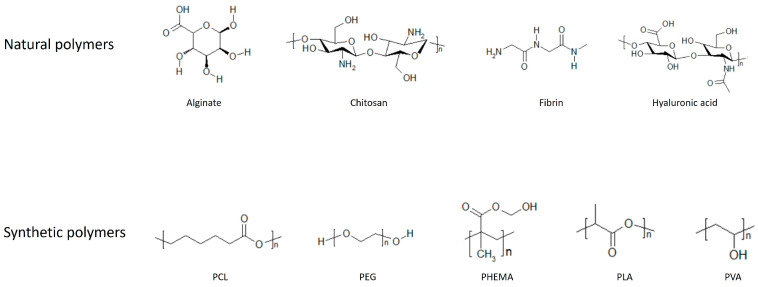
**Chemical structures of commonly used natural and synthetic materials**. PCL: polycaprolactone, PEG: poly(ethylene glycol), PHEMA: poly(2-hydroxyethyl methacrylate), PLA: poly(lactic acid), PVA: poly(vinyl alcohol).

Figure 1 shows chemical structures of the most commonly used materials for the synthesis of hydrogels.

Because of their properties and the ability to be delivered with minimally invasive procedures, hydrogels have been studied for the treatment of cardiovascular diseases.

## 2. Cardiac Repair and Regeneration

Myocardial infarction (MI) occurs when blood flow does not reach the heart properly due to an obstruction of coronary arteries, leading to damage and necrosis of the cardiac muscle because of lack of oxygen [30]. Afterwards, local tissue repair is activated. Repair is a biological process that involves the deposition of fibrous tissue to address the defect created by the wound. In fact, after MI damage, the necrotic myocardium is replaced by a fibrotic scar while the myocardium adjacent to the infarcted area becomes thinner. In addition, ECM degradation and loss of cardiomyocytes occur, and remodeling of the left ventricle (LV) takes place, contributing to decrease ventricular function [31,32,33].

There are different pathways implicated in cardiac repair: TGF-β signaling is involved in apoptosis, hypertrophic and fibrotic remodeling of the heart, inflammation, and ECM deposition; Wnt signaling is predominantly involved in the progression of cardiac fibrosis via interaction with TGF-β signaling; the renin-angiotensin-aldosterone system is implicated in the activation of cardiac fibrosis [34].

The damage caused by a heart attack leads to a permanent loss of cardiac tissue in adult mammals. Regeneration is a complicated process that involves the restitution of tissue components, and its aim is therefore to obtain a tissue with characteristics indistinguishable from the original one. Adult human cardiomyocytes are terminally differentiated and have virtually no regenerative capacity, making it difficult to restart cardiomyocyte proliferation. However, some pathways implicated in the reactivation of the cellular cycle of cardiomyocytes have been identified: Hippo-Yap is involved in the proliferation, migration, and apoptosis of cardiomyocytes, in fact, it has been observed that its deficiency improves the regeneration of cardiomyocytes in adult mice; Notch is an important pathway in cardiac generation in zebrafish, and it regulates the maturation of the endocardium and promotes the proliferation of cardiomyocytes; Nrg1 induces cell cycle re-entry and cardiomyocyte division in adult mice [35]. The regeneration of cardiomyocytes has also been observed in humans [36], but as this is insufficient to restore the contractile function of the damaged heart, it is therefore important for patients to implement regenerative therapies.

**Figure 2:**
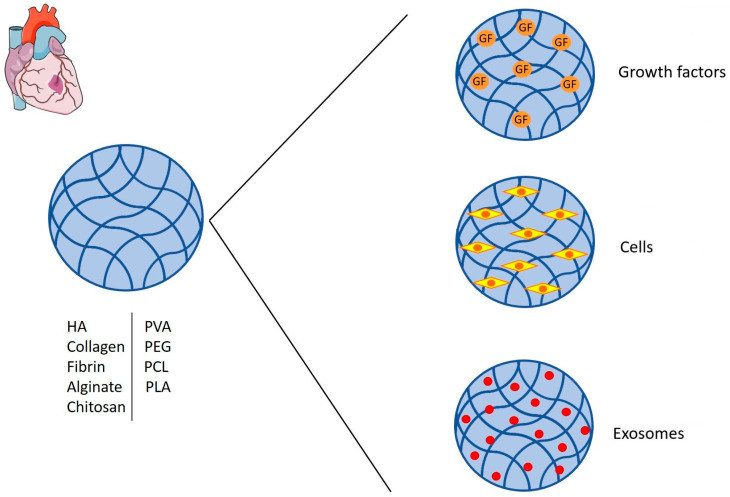
**Hydrogel approaches for MI treatment.** In the figure, the different therapeutic approaches investigated to restore cardiac functions following MI are depicted. This figure was created by adapting a Servier Medical Art template [licensed under Creative Commons Attribution 3.0 Unported License www.smart.servier.com (accessed on 12 December 2023)].

Therapeutic approaches for MI are directed towards the stabilization or improvement of myocardial function; in this context, hydrogels have been used to aid in ventricular remodeling and to improve the delivery and viability of molecules and cells to the infarcted area to enhance cardiomyocyte survival and improve cardiac functions (Figure 2).

### 2.1. Ventricular Wall Thickening

After MI, the residual normal myocardium implements compensatory mechanisms to cope with the decline in cardiac function. The loss of cardiomyocytes triggers a remodeling process that consists of several steps, i.e., infarct expansion, wall dilation, hypertrophy, and collagen scarring. Infarct expansion results in LV remodeling, which is an alteration in ventricular structure. The ventricular mass increases to cope with the thinning of the myocardium adjacent to the infarcted area and a ventricular dilation occurs to preserve the blood volume [37,38]. Therefore, LV remodeling involves ECM degradation, infarcted zone expansion, and ventricular expansion. Unfortunately, to date, this is associated with poor clinical outcomes [39].

Hydrogel injection therapy consists of injecting biomaterials in the infarcted myocardium and has been investigated as a strategy to thicken the wall of an infarcted area to provide support to the LV wall and reduce its remodeling, aiming to restore myocardial mechanical properties.

Zhu et al. have reviewed the advances made in biomaterials injection therapy and discussed a possible direction for future research. Since the introduction of this concept, there have been some promising results that have advanced in clinical trials. To achieve clinical success, it is necessary to understand the mechanism by which this type of therapy promotes the restoration of cardiac functions and, therefore, how to improve the development of materials to obtain better results. In addition, attention must be paid to the administration methods; imaging techniques can be helpful to understanding the best areas in which to perform the injection and, thus, optimize the outcome for a patient [40].

### 2.2. Growth Factors and Cells Delivery

The full restoration of heart function occurs through heart transplantation; however, this treatment has limits regarding the availability of donors that does not meet the demand. Therefore, studies have focused on alternatives to address the problem, such as the development of hydrogels for cell and molecule delivery to cope with the loss of cardiomyocytes and restore cardiac functions. The direct administration of cells and molecules has some problems, such as low half-life, low cell survival, non-specific localization, and low cell concentration to the target area; the use of hydrogels has been helpful in limiting degradation and improving cell survival and delivery to the area of interest [41,42].

**Growth factors.** Vascular endothelial growth factor (VEGF) is an important proangiogenic factor, and it promotes endothelial cells survival, proliferation, and migration [43]. Hydrogels capable of releasing VEGF for the treatment of MI were therefore developed to improve angiogenesis and cardiac function. A hydrogel comprised of PEG and fibrinogen loaded with VEGF was injected in a rat model of MI, obtaining the release of VEGF for 30 days, which induced the proliferation and migration of endothelial cells, a reduction in cardiac remodeling, and an improvement in ventricular function [44]. Another group synthesized a hydrogel formed from biodegradable dextran chains grafted with hydrophobic poly-(e-caprolactone)-2-hydroxyethyl methacrylate (PCL-HEMA) chains and PCL (polycaprolactam)-grafted polysaccharide chains into the PNIPAAm network loaded with VEGF165. They achieved VEGF release for up to 30 days, as well as improvement in heart function, angiogenesis promotion, and a decrease in the infarcted area [45]. Wu et al. have produced an alginate hydrogel loaded with VEGF and silk fibroin (SF) microspheres containing bone morphogenetic protein 9 (BMP9), which was linked with a reduction in myocardial fibrosis [46] when it was injected in an MI mice model. VEGF was released more rapidly to stimulate angiogenesis at an early stage, while BMP9 was liberated slowly to inhibit fibrosis in the long-term stage, resulting in improved cardiac function [47].

Other factors have also been used, such as engineered stromal cell-derived factor-1α (ESA), a synthetic analogue of stromal cell-derived factor 1 (SDF1), which improves mechanical function and decreases ventricular remodeling after MI [48]. A HA hydrogel was used in an MI rat model for the delivery of ESA; after hydrogel injection, ESA was released for more than 28 days, accomplishing angiogenesis stimulation and the maintenance of LV geometry [49]. Recently, Perez-Estenaga et al. developed a collagen-on-collagen scaffold to deliver SDF1 in a rat MI model. The scaffold was successfully integrated into the heart and its therapeutic effect was observed through the improvement in cardiac functions, the reduction in heart stiffness, and the pro-angiogenic effect [50].

Insulin-like growth factor 1 (IGF-1) is involved in the survival of cardiomyocytes and low levels of IGF-1 are associated with CVD development [51,52]; furthermore, it protects cardiomyocytes from oxidative stress [53]. IGF-1 was encapsulated in SF microspheres and loaded in an alginate-based hydrogel. The hydrogel was injected in rats after MI, and IGF-1 release occurred for 28 days, resulting in increased cardiac function, a reduction in fibrosis, and lower cardiomyocyte apoptosis [54]. Fang et al. have developed a hydrogel for the codelivery of IGF-1 and 6-bromoindirubin-3-oxime (BIO), an inhibitor of glycogen synthase kinase-3 which promotes cardiomyocytes proliferation [55]. The hydrogel is composed of oxidized alginate and gelatin nanoparticles in which IGF-1 and BIO are incapsulated; when injected in rats after MI, improvements in myocardial functions and cardiomyocyte proliferation were achieved [56].

Myeloid-derived growth factor (MYDGF) has been shown to promote cardiomyocytes survival while reducing scarring and improving ventricular functions in rats after MI [57]. Yuan et al. have developed an injectable citrate-based polyester hydrogel for the local sustained delivery of MYDGF in the heart after MI, which resulted in improved cardiac morphology and functionality, increased angiogenesis, and improved cardiomyocytes survival [58].

Basic fibroblast growth factor (bFGF) is another angiogenic factor that induces the proliferation of smooth muscle cells (SMCs), endothelial cells, and fibroblasts [59]. bFGF was delivered to rats’ infarcted hearts using a thermosensitive, fast-gelatinization, glutathione (GSH)-modified collagen hydrogel (Gel-GSH), thereby inducing the release of bFGF for 28 days, increasing wall thickness, decreasing cardiac fibrosis, and enhancing vascularization [60]. Fan et al. have developed a NIPAAM-based injectable hydrogel to promote angiogenesis with bFGF and to inhibit cardiac remodeling by targeting the upregulated matrix metalloproteinases 2/9 (MMP-2/9), which are responsible for the degradation of the ECM that contributes to LV dilation. This resulted in improved cardiac function, increased vascularization, and improved myocardial remodeling [61]. Furthermore, Fu et al. have developed a disulfide cross-linked chitosan loaded with bFGF, and after its injection into an in vivo rat MI model it was shown that the hydrogel improved left ventricular function, reduced fibrotic area, reduced myocyte apoptosis, and promoted angiogenesis [62].

Rosmarinic acid (RA) has also been taken into consideration for the treatment of MI. It is a polyphenolic antioxidant that has shown anti-inflammatory, anti-apoptotic, and anti-fibrotic properties. Zhang et al. have recently incapsulated polydopamine-RA nanoparticles in a hydrogel composed of gelatin, oxidized xanthan gum grafted with 3-aminophenylboronic acid (OXP), and dopamine-grafted gelatin (GD), which they injected in an in vivo rat MI model. The hydrogel was shown to promote angiogenesis, improve cardiac functions, improve electrical conduction in the infarcted area, improve ventricular wall thickening and reduce the fibrotic area [63].

Angiopoietin-like 4 (ANGPTL4) is a protein with anti-inflammatory properties, and it promotes the migration of endothelial cells and angiogenesis. Lee et al. have incorporated ANGPTL4 into a cardiac patch composed of gelatin and dextran-aldehyde, and they studied its effect in a rat MI model. The painted hydrogel covered the entire LV, including the infarcted area, and was shown to improve cardiac function, reduce the fibrotic area, enhance angiogenesis, and suppress the presence of inflammatory macrophages [64].

Recently, Hu et al. have developed an injectable hydrogel composed of phenylboronic acid-grafted carboxymethyl cellulose (CMC-BA) and PVA for the delivery of curcumin and recombinant humanized collagen type III (rhCol III) in the infarcted area in a rat MI model. The hydrogel improved cardiac function, enhanced LV wall thickness, reduced infarct size, reduced cardiomyocyte apoptosis, and decreased inflammation [65].

**Cells.** Stem cells are undifferentiated cells with characteristics such as self-renewal (ability to proliferate extensively), clonality (monotypic expansion from a single cell), and potency (potential to differentiate into different cell types) [66]. For this reason, they have been largely studied in regenerative medicine, and for MI their role has been investigated for cardiomyocyte regeneration. Different types of stem cells have been considered: mesenchymal stem cells (MSCs), embryonic stem cells (ESCs), and induced pluripotent stem cells (iPSCs).

Using a fibrin-based hydrogel, MSCs were delivered in a rat model of acute MI. The use of the hydrogel allowed for local cell retention, increased cell survival, and minimized MSC distribution in other organs [67]. Levit et al. have used an alginate hydrogel to encapsulate MSCs. The hydrogel was then implanted in a PEG hydrogel patch and delivered in rats after the induction of MI, achieving cell retention in the myocardium, a reduction in scarring, and improved cardiac function [68]. A self-setting silanized hydroxypropyl methylcellulose (Si-HPMC) hydrogel was also investigated for MSC delivery in infarcted rats’ hearts, obtaining positive results for short- and mid-term effects in LV remodeling and in the preservation of endocardial myocytes [69]. Another type of hybrid hydrogel was used for the delivery of MSCs in an MI rat model. It was based on thiolated collagen (Col-SH) and multiple acrylate-containing oligo(acryloyl carbonate)-b-poly(ethylene glycol)–oligo(acryloyl carbonate) (OAC-PEG-OAC) copolymers, and resulted in a significantly reduced infarct size and increased wall thickness [70].

Wu et al. have recently studied the treatment of MI via the co-culture of MSCs and cardiomyoblasts (H9C2) cells on gold-loaded chitosan/silk fibroin hydrogel (Au@Ch-SF) in a rat MI model. A regrowth of cardiac muscle fibers, a decrease in fibrotic area, a decrease in apoptosis, and an improvement in cardiac functions have been demonstrated [71].

MSCs were also encapsulated in a cardiac patch composed of cardiogel and a chitosan scaffold. The use of the patch was studied in a rat MI model, and improved cell retention and survival, an improvement in cardiac functions, and an increase in wall thickening were observed [72].

Lately, Chen et al. have developed Col-Tgel, a collagen-based hydrogel, in which adipose-derived mesenchymal stem cells (ADSCs) were engrafted. The hydrogel was injected in mice after MI, resulting in ADSC survival and retention, a reduction in myocardial fibrotic area, and improved cardiac function [73]. Furthermore, Lyu et al. have designed a HA-based injectable hydrogel encapsulated with human mesenchymal stem cells (hMSCs) that was inserted in rats after MI. The treated group showed decreased fibrosis, increased infarct wall thickness, and a promotion of angiogenesis [74].

Umbilical cord mesenchymal stem cells (UCMSCs) have recently been investigated as a potential treatment of MI. UCMSCs were introduced into a hydrogel composed of gelatin methacrylate (GelMA) and oxidized dextran (ODEX), which was then injected into a rat MI model. In vivo experiments demonstrated that the hydrogel significantly reduced the infarcted area, preserved LV wall thickness, inhibited vascular remodeling, and decreased cardiomyocytes apoptosis [75].

Human bone marrow mesenchymal stem cells (hBMSCs) have also been considered for the treatment of MI. Indeed, Karimi Hajishoreh et al. have developed an electroactive hydrogel composed of reduced graphene oxide (rGO) and alginate (ALG), in which hBMSCs were encapsulated. The hydrogel was then injected into an in vivo rat MI model. The hydrogel injection improved LV function and wall thickness, induced angiogenesis, and decreased fibrotic area [76].

Recently, Hong et al. used Gel-MA for the delivery of human endothelial colon-forming cells (ECFCs) and MSCs in a mouse MI model. The Gel-MA allowed for enhancement of the retention of cells at the injection site and an improvement in cardiac functions; additionally, a decrease in fibrosis and improved revascularization were observed [77].

Mouse embryonic stem cells (mESCs) were encapsulated in a biodegradable hydrogel composed of oligo[poly(ethylene glycol) fumarate] (OPF) that was then injected into the LV wall of rats one week after MI induction. The treatment led to a reduction in infarct size, lower MMP-2 and MMP-9 levels, and LV function improvement [78]. Tan et al. have investigated which biomaterial is the best for the delivery of human embryonic stem cell-derived cardiomyocytes (hESC-CMs) between Matrigel, alginate, and hyaluronate. The different hydrogels were engrafted with hESC-CMs and injected into the myocardium of rat MI models. The alginate hydrogel effectively prevented LV remodeling; however, hyaluronate showed the best effect in delaying LV remodeling and improving cardiac functions [79].

iPSCs can be used as a source to obtain autologous cardiomyocytes. The role of iPSC-derived cardiomyocytes (iPSC-CMs) was investigated with the aim to restore cardiac function in a rat model of MI. A peptide-modified hydrogel was used for cell delivery, enhancing the survival of the iPSC-CMs; in addition, an improvement in LV function was achieved [80]. Li et al. incapsulated iPSCs in a folic acid (FA) hydrogel and then injected it into MI mice hearts. Cell retention and increased cardiac function were accomplished, with decreased collagen levels and the promotion of neovascularization also observed [81].

A detailed review about the use of cells in cardiac regeneration can be found here [82].

**Exosomes.** In recent years, the role of exosomes has also been investigated in regenerative medicine; they are extracellular vesicles of endosomal origin that contain proteins and RNA molecules with potential cardioprotective properties [83]. Han et al. have investigated the potential of human umbilical cord mesenchymal stem cell-derived exosomes (UMSC-Exos) in improving heart function in an MI rat model. The UMSC-Exos were loaded on a peptide-based hydrogel and injected in rats after MI, inducing a decrease in fibrosis and inflammation and an improvement in cardiac functions [84].

Recently, Yan et al. incorporated human endometrial mesenchymal stem cell (hEMSC)-derived exosomes (hEMSC-Exos) into poly-pyrrole-chitosan (PPY-CHI) hydrogel. It was observed that the PPY-CHI/hEMSC-Exos reduced apoptosis and promoted angiogenesis in a rat MI model. Furthermore, the in vivo injection of PPY-CHI/hEMSC-Exos allowed for thickening of the ventricular wall, reduction in the fibrotic area, improvement in functional parameters, and reduction in post-MI arrhythmia [85].

**Hybrid approaches.** The combination of growth factors and cells delivered with hydrogels has also been studied. A hydrogel composed of HA and PEG and loaded with Wharton’s jelly mesenchymal stem cells (HWJMSCs) and IGF-1 was injected in a rabbit model of MI. The administration of the hydrogel resulted in enhanced angiogenesis, reduced inflammation, smaller infarct size and improved cardiac functions [86].

Recently, Liang et al. have developed a hydrogel composed of partially oxidized alginate cross-linked with tetraaniline (TA) nanoparticles and engrafted with 2-aminopyridine-5-thiocarboxamide (APTC) and adipose-derived stem cells (ADSCs). APTC is used as a source of hydrogen sulfide (H2S), which has anti-inflammatory effects, provides protection against oxidative stress, and has been shown to reduce infarct size. The hydrogel was injected in rats after MI and was seen to improve LV functions and decrease the fibrotic area [87].

Moreover, a hydrogel composed of decellularized porcine extracellular matrix containing SDF-1 and cardiomyocytes was recently developed, and in vitro studies have confirmed its biocompatibility and antiapoptotic ability; meanwhile, in vivo studies have confirmed its roles in improved cardiomyocyte retention and a better intercellular communication, which are important for maintaining normal cardiac rhythm. Furthermore, the hydrogel promoted angiogenesis, and it was also shown to reduce the area of fibrosis in the infarcted area [88].

Table 1 summarizes the studies on the use of hydrogels for the treatment of MI.

**Figure 3:**
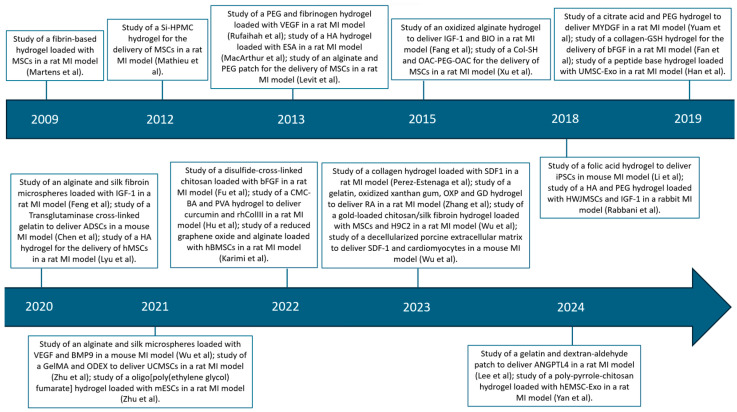
**Timeline of studies using hydrogel for the delivery of growth factors, cells, and exosomes for the treatment of MI [44,47,49,50,54,56,58,60,62,63,64,65,67,68,69,70,71,73,74,75,76,81,84,85,86,88].**

In Figure 3 the studies are depicted in chronological order.

## 3. Arterial Repair

### 3.1. Hydrogel-Coated Coils in Intracranial Aneurysms

Intracranial aneurysms (IAs) are localized dilations of the cerebral arteries’ walls and are prone to rupture, resulting in bleeding. They can be treated with surgical or endovascular methods. Surgical treatment is called clipping, which consists of exposing the aneurysm via craniotomy and excluding it from the circulation using clips. However, this is an invasive technique, so the endovascular method is preferred. This consists of delivering platinum coils within the aneurysmal sac to induce the formation of blood clots to exclude the aneurysm from the circulation [89]. Even though the endovascular treatment is less invasive, it is linked with a higher rate of aneurysm recurrence and retreatment; therefore, there is a need to improve this method. In this regard, hydrogel-coated coils have been studied. Recently, the work of Xue et al. 2018 compared trials which has been carried out to investigate the value of endovascular treatment with hydrogel-coated coils and to highlight potential factors that could affect their safety and efficacy. Their analysis brought to light that endovascular treatment with hydrogel-coated coils is useful for preventing mid-term recurrence and residual aneurysm, but not for complete occlusion. These second-generation hydrogel-coated coils show some potential in mid-term complete occlusion, so future research can be directed to exploring their possible therapeutic effect for IA treatment [90].

### 3.2. Abdominal Aortic Aneurysm (AAA)

AAA is a pathological dilation of a segment of the abdominal aorta to greater than 1.5 times its normal size; an aortic diameter of 3 cm is already considered aneurysmatic [91,92]. According to the American and European guidelines, the indication for AAA treatment is given when an AAA reaches diameters of 5.5 and 5.2 in men and women, respectively. If left untreated, it is estimated that one-third of these aneurysms will eventually rupture, with an overall mortality rate of up to 90% [93].

Endovascular aortic aneurysm repair (EVAR) is currently the most common method for the elective treatment of AAAs. EVAR involves the use of metal stent grafts covered with impermeable (polytetrafluoroethylene or polyester) fabric to cover the aorta. The sealing of the stent relative to the aortic wall at the proximal and distal ends of the aneurysm removes the aneurysm sac from systemic circulation, thereby preventing subsequent rupture [94]. The success of this intervention is limited due to the occurrence of endoleaks, which describe the continuous flow of blood into the aneurysmal sac outside the stent graft, with failure of complete exclusion of the aneurysm [95]. The standard treatment for endoleaks is the use of metallic coils injected into the aneurysm sac to induce coagulation and thereby restore aneurysm exclusion from systemic circulation.

**Figure 4:**
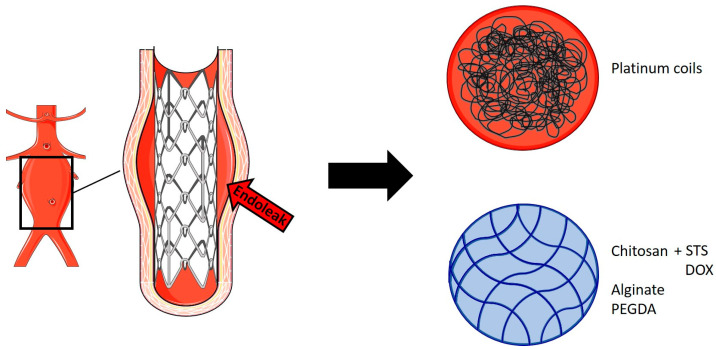
**EVAR complication and treatment approaches.** After treatment of AAA using EVAR, the occurrence of endoleaks can take place, resulting in the failure of the complete exclusion of an aneurysm from systemic circulation. The standard treatment for endoleaks is the use of metallic coils, which are injected into the aneurysm sac to induce coagulation and thus restore the exclusion of the aneurysm. However, the use of hydrogels could represent an effective approach for the treatment of endoleaks. This figure was created by adapting a Servier Medical Art templates [licensed under Creative Commons Attribution 3.0 Unported License www.smart.servier.com (accessed on 12 December 2023)].

Hydrogels have been studied for the treatment and prevention of endoleaks, although there are still few published studies (Figure 4). Fatimi et al. have developed an injectable chitosan hydrogel containing sodium tetradecyl sulfate (STS), which is a sclerosing agent, for the embolization of aneurysms. The hydrogel was tested in vivo in a canine bilateral iliac aneurysm model reproducing persistent endoleaks after EVAR; consequently, the intervention resulted in the prevention of endoleaks after three months in the group treated with chitosan-STS hydrogel [96].

Barnett et al. have developed two different types of hydrogels for the treatment of fusiform and saccular aneurysms, and both were investigated in vitro. EmboGel is an alginate-based hydrogel, and its use has been studied in a silicone model of AAA, while UltraGel is a poly(ethylene-glycol)-diacrylate (PEGDA)-based hydrogel in which polymerization was achieved by UV light, and its use has been investigated on a glass model of saccular aneurysm. EmboGel has been demonstrated to fill and occlude the aneurysm sac that surrounds a stent, with the potential to prevent endoleaks; meanwhile, UltraGel has been shown to be able to achieve occlusion of a saccular aneurysm from the flow [97].

Recently, Zehtabi et al. have explored the application of a chitosan-based hydrogel containing doxycycline (DOX) for the treatment of endoleaks. DOX is a sclerosant agent which promotes thrombosis and inhibits MMP activity, decreasing AAA progression. Preliminary in vivo studies were conducted on pigs, in which the hydrogel was injected after bilateral embolization of the caudal branch of the renal artery. DOX was released with an initial surge in the first 24 h and then with a slow and continuous release in the following week, resulting in the embolization of blood vessels, although higher DOX concentrations may be needed to avoid recanalization after embolization [98].

Table 2 summarizes the studies on the use of hydrogels for the treatment of endoleaks.

## 4. Conclusions

Hydrogels are polymeric networks widely used for biomedical applications, as they are biocompatible, hydrophilic, and can be modified according to their application. In the context of cardiovascular diseases, they could represent an effective therapeutic strategy. For the treatment of MI, they have been studied to prevent and limit LV remodeling, restore lost cardiomyocytes, and, therefore, recover cardiac functions; their use has been effective in providing growth factors and cells in the infarcted site since, unlike direct administration, it has been possible to preserve and obtain a controlled release, preventing their degradation and improving their localization. Few studies have been directed to the treatment of arterial aneurysms. For IA, hydrogel applications have been investigated for their occlusion and to prevent their rupture, while for AAAs they have been studied to prevent endoleaks after EVAR, which is the standard AAA treatment. Overall, there are still few studies that have achieved effective results; therefore, additional investigations are needed to advocate the clinical efficacy of hydrogels in the cardiovascular field.

## Figures and Tables

**Table 1:** **Summary of studies using hydrogels loaded with growth factors, cells, and exosomes for the treatment of MI.**

Author/Year	Hydrogel Composition	Loading Composition	Reference
Rufaihah et al., 2013	PEG and fibrinogen	VEGF	[44]
Zhu et al., 2015	Biodegradable dextran chains grafted with hydrophobic PCL-HEMA chains and PCL-grafted polysaccharide chains into the PNIPAAm network	VEGF165	[45]
Wu et al., 2021	Alginate and silk microspheres	VEGF and BMP9	[47]
MacArthur et al., 2013	Hyaluronic acid	ESA	[49]
Perez-Estenaga et al., 2023	Collagen	SDF1	[50]
Feng et al., 2020	Alginate and silk fibroin microspheres	IGF-1	[54]
Fang et al., 2015	Oxidized alginate and gelatin nanoparticles	IGF-1 and BIO	[56]
Yuan et al., 2019	Citrate acid and PEG	MYDGF	[58]
Fan et al., 2019	Collagen-GSH	bFGF	[60]
Fan et al., 2020	NIPAAm, HEMA, and AOLA	bFGF	[61]
Fu et al., 2022	Disulfide cross-linked chitosan	bFGF	[62]
Zhang et al., 2023	Gelatin, oxidized xanthan gum, OXP, and GD	RA	[63]
Lee et al., 2024	Gelatin and dextran-aldehyde	ANGPTL4	[64]
Hu et al., 2022	Phenylboronic acid-grafted carboxymethyl cellulose (CMC-BA) and PVA	Curcumin and rhColIII	[65]
Martens et al., 2009	Fibrin	MSCs	[67]
Levit et al., 2013	Alginate and PEG	MSCs	[68]
Mathieu et al., 2012	Silanized hydroxypropyl methylcellulose	MSCs	[69]
Xu et al., 2015	Thiolated collagen and OAC-PEG-OAC	MSCs	[70]
Wu et al., 2023	Gold-loaded chitosan/silk fibroin hydrogel	MSCs and cardiomyoblasts	[71]
Sharma et al., 2022	Cardiogel and chitosan	MSCs	[72]
Chen et al., 2020	Transglutaminase cross-linked gelatin	ADSCs	[73]
Lyu et al., 2020	Hyaluronic acid	hMSCs	[74]
Zhu et al., 2021	GelMA and ODEX	UCMSCs	[75]
Karimi Hajishoreh et al., 2022	Reduced graphene oxide and alginate	hBMSCs	[76]
Hong et al., 2023	GelMA	Human endothelial colon-forming cells and MSCs	[77]
Wang et al., 2021	Oligo[poly(ethylene glycol) fumarate]	mESCs	[78]
Tan et al., 2020	Matrigel, alginate, and hyaluronate	hESC-CMs	[79]
Wang et al., 2015	PEG-PCL conjugated with a collagen-binding peptide (SYIRIADTNIT)	iPSC-CMs	[80]
Li et al., 2018	Folic acid	iPSCs	[81]
Han et al., 2019	Peptide based	UMSC-Exo	[84]
Yan et al., 2024	Poly-pyrrole-chitosan	hEMSC-Exo	[85]
Rabbani et al., 2018	Hyaluronic acid and PEG	HWJMSCs and IGF1	[86]
Liang et al., 2019	Partially oxidized alginate cross-linked with tetraaniline nanoparticles	APTC and ADSCs	[87]
Wu et al., 2023	Decellularized porcine extracellular matrix	SDF-1 and cardiomyocytes	[88]

**Table 2:** **Summary of studies using hydrogels for the treatment of endoleaks.**

Author/Year	Hydrogel Composition	Loading Composition	Reference
Fatimi et al., 2012	Chitosan	STS	[96]
Barnett et al., 2009	EmboGel and Ultragel		[97]
Zehtabi et al., 2017	Chitosan	DOX	[98]

## Abbreviations

AAA: abdominal aortic aneurysm; ADSCs: adipose-derived mesenchymal stem cells; ALG: alginate; ANGPTL4: angiopoietin-like 4; APTC: 2-aminopyridine-5-thiocarboxamide; bFGF: basic fibroblast growth factor; BIO: 6-bromoindirubin-3-oxime; BMP9: bone morphogenetic protein 9; CMC-BA: phenylboronic acid-grafted carboxymethyl cellulose; Col-SH: thiolated collagen; CVD: cardiovascular disease; DOX: doxycycline; ECFCs: human endothelial colony-forming cells; ECM: extracellular matrix; ESA: engineered stromal cell-derived factor-1α; ESCs: embryonic stem cells; EVAR: endovascular aortic aneurysm repair; FA: folic acid; Gel-GSH: glutathione-modified collagen hydrogel; GD: dopamine-grafted gelatin; GelMA: gelatin methacrylate; GSH: glutathione; H2S: hydrogen sulfide; HA: hyaluronic acid; hBMSCs: human bone marrow mesenchymal stem cells; hEMSC: human endometrial mesenchymal stem cell; hEMSC-Exos: human endometrial mesenchymal stem cell (hEMSC)-derived exosomes; hESC-CMs: human embryonic stem cell-derived cardiomyocytes; hMSCs: human mesenchymal stem cells; HWJMSCs: Wharton’s jelly mesenchymal stem cells; IA: intracranial aneurysm; IGF-1: insulin like growth factor 1; iPSC-CMs: iPSCs-derived cardiomyocytes; iPSCs: induced pluripotent stem cells; LV: left ventricle; MA: methacrylic anhydride; mESCs: mouse embryonic stem cells; MI: myocardial infarction; MMP-2: metalloproteinase 2; MMP-9: metalloproteinase 9; MSCs: mesenchymal stem cells; MYDGF: myeloid-derived growth factor; OAC-PEG-OAC: oligo(acryloyl carbonate)-b-poly(ethylene glycol)–oligo(acryloyl carbonate); ODEX: oxidized dextran; OPF: oligo[poly(ethylene glycol) fumarate]; OXP: oxidized xanthan gum grafted with 3-aminophenylboronic acid; PCL: polycaprolactone; PCL-HEMA: poly-(e-caprolactone)-2-hydroxyethyl methacrylate; PEG: poly(ethylene glycol); PEGDA: poly(ethylene-glycol)-diacrylate; PHEMA: poly(2-hydroxyethyl methacrylate); PLA: poly(lactic acid); PNIPAAm: poly(N-isopropylacrylamide); PPY-CHI: poly-pyrrole-chitosan; PVA: poly(vinyl alcohol); PVAc: polyvinyl acetate; RA: rosmarinic acid; rhCol III: recombinant humanized collagen type III; rGO: reduced graphene oxide; SDF1: stromal cell-derived factor 1; SF: silk fibroin; Si-HPMC: silanized hydroxypropyl methylcellulose; SMCs: smooth muscle cells; STS: sodium tetradecyl sulfate; TA: tetraaniline; UCMSCs: umbilical cord mesenchymal stem cells; UMSC-Exo: umbilical cord mesenchymal stem cell-derived exosomes; VEGF: vascular endothelial growth factor.
